# The action of a negative allosteric modulator at the dopamine D_2_ receptor is dependent upon sodium ions

**DOI:** 10.1038/s41598-018-19642-1

**Published:** 2018-01-19

**Authors:** Christopher J. Draper-Joyce, Ravi Kumar Verma, Mayako Michino, Jeremy Shonberg, Anitha Kopinathan, Carmen Klein Herenbrink, Peter J. Scammells, Ben Capuano, Ara M. Abramyan, David M. Thal, Jonathan A. Javitch, Arthur Christopoulos, Lei Shi, J. Robert Lane

**Affiliations:** 1 Drug Discovery Biology, Monash Institute of Pharmaceutical Sciences, Monash University, 399 Royal Parade, Parkville, VIC 3052 Australia; 2 Medicinal Chemistry, Monash Institute of Pharmaceutical Sciences, Monash University, 399 Royal Parade, Parkville, VIC 3052 Australia; 3 Computational Chemistry and Molecular Biophysics Unit, National Institute on Drug Abuse Intramural Research Program, National Institutes of Health, 333 Cassell Drive, Baltimore, Maryland 21224 United States; 4 Departments of Psychiatry and Pharmacology, New York State Psychiatric Institute, New York, New York 10032 United States; 5 College of Physicians and Surgeons, Columbia University, and Division of Molecular Therapeutics, New York State Psychiatric Institute, New York, New York 10032 United States; 6 Present Address: Tri-Institutional Therapeutics Discovery Institute, 413 E 69th St, New York, NY 10021 United States

## Abstract

Sodium ions (Na^+^) allosterically modulate the binding of orthosteric agonists and antagonists to many class A G protein-coupled receptors, including the dopamine D_2_ receptor (D_2_R). Experimental and computational evidences have revealed that this effect is mediated by the binding of Na^+^ to a conserved site located beneath the orthosteric binding site (OBS). SB269652 acts as a negative allosteric modulator (NAM) of the D_2_R that adopts an extended bitopic pose, in which the tetrahydroisoquinoline moiety interacts with the OBS and the indole-2-carboxamide moiety occupies a secondary binding pocket (SBP). In this study, we find that the presence of a Na^+^ within the conserved Na^+^-binding pocket is required for the action of SB269652. Using fragments of SB269652 and novel full-length analogues, we show that Na^+^ is required for the high affinity binding of the tetrahydroisoquinoline moiety within the OBS, and that the interaction of the indole-2-carboxamide moiety with the SBP determines the degree of Na^+^-sensitivity. Thus, we extend our understanding of the mode of action of this novel class of NAM by showing it acts synergistically with Na^+^ to modulate the binding of orthosteric ligands at the D_2_R, providing opportunities for fine-tuning of modulatory effects in future allosteric drug design efforts.

## Introduction

Dopamine receptors (DRs) are class A G-protein coupled receptors (GPCRs) implicated in the biology and treatment of a number of central nervous system disorders^1^. Drug development at these receptors has focused upon targeting the orthosteric site where dopamine binds. However, the advantages of targeting allosteric sites have begun to be explored for many GPCRs. This includes the dopamine D_2_ receptor (D_2_R), for which drug-like positive (PAM) and negative (NAM) allosteric modulators have recently been identified^2–7^. SB269652 (**1**) is the first small molecule NAM of the D_2_R despite having a structure similar to many competitive antagonists of the D_2_-like DRs^5^. This structure is comprised of a tetrahydroisoquinoline (THIQ) primary pharmacophore (PP) connected by a cyclohexylene linker to a lipophilic 1*H*-indole-2 carboxamide secondary pharmacophore (SP). Truncated derivatives of SB269652 containing the THIQ PP, which is protonated at physiological pH, act in a competitive manner with dopamine. Our previous modelling studies predicted that the basic tertiary amine within the THIQ PP of SB269652 forms a salt bridge with Asp^3.32^ (Ballosteros and Weinstein numbering system)^8^ in the orthosteric binding site (OBS), while the 1*H*-indole-2-carboxamide SP of SB269652 extends away from the OBS to interact with residues in a secondary binding pocket (SBP) between the extracellular ends of transmembrane segments (TMs) 2 and 7^2^. To reconcile an orthosteric mode of engagement with an allosteric mechanism of action, we proposed that SB269652 acts to engage one protomer of a D_2_R dimer in a bitopic binding mode to modulate dopamine binding and function in the adjacent protomer^2^.

The modulatory effects of sodium ions (Na^+^) on orthosteric ligand binding at class A GPCRs has been well characterised^9–14^. Recently, high resolution structures of the adenosine A_2A_ receptor (A_2A_AR)^15^ the β_1_ adrenergic receptor^16^, protease activated receptor^17^, the δ-opioid receptor^18^, and the D_4_R^19^, revealed a Na^+^-binding site below the orthosteric pocket in which a Na^+^ is coordinated by the side chain oxygen atoms of Asp^2.50^, Ser^3.39^, and three water molecules. Molecular dynamics (MD) studies of the A_2A_AR show that the presence of Na^+^ in this pocket inhibits conformational movement of TM7 toward TM3 at the intracellular side, stabilising an “inactive” conformation. Thus, the absence of Na^+^ is associated with the active conformation of the receptor, which has a greater dynamic flexibility in the movement of Trp^6.48^ and Asn^7.45^ ^20,21^.

Physiologically relevant concentrations of Na^+^ have been shown to decrease agonist affinity for the D_2_R while enhancing the affinity for some classes of antagonists^22,23^. Substituted benzamides, including eticlopride and sulpiride, display increased affinity in the presence of Na^+^, whereas spiperone is insensitive and the affinity of zotepine is decreased^24^. Substitution of Asp^2.50^ of the D_2_R with an uncharged residue reduces the affinity of some antagonists^11,12^. Our previous docking and MD studies provided insight into the mechanism behind the distinct Na^+^ sensitivities of these antagonists, revealing an allosteric interaction network that propagates cooperative effects from the Na^+^ binding site to the OBS^24^. In particular, Na^+^ binding stabilised an interaction between the side chains of Asp^3.32^ and Tyr^7.43^. In the Na^+^-unbound condition, the less optimal binding modes of the Na^+^ sensitive antagonists are correlated with the weakening of this hydrogen bond interaction. Another study revealed that Na^+^ binding to this conserved site was important for the subtype selectivity of D_4_R-selective agonists and antagonists through modulation of the interaction of the SP of such ligands to a residue within the SBP^25^. Using MD simulations, Selent and coworkers proposed that Na^+^ enters the D_2_R via the extracellular side of the receptor, moving along a network of negatively charged residues including Glu95^2.65^ before entering the Na^+^-binding site coordinated by Asp80^2.50^. This Na^+^ binding was proposed to lock the rotamer toggle switch Trp386^6.48^ in the inactive state^26^.

In addition to these effects on orthosteric ligand binding, Na^+^ ions have been shown to modulate the action of BMS986122, a PAM of the µ-opioid receptor, which was shown to exert its effect through disruption of the Na^+^ binding site^27^. This study, as well as the critical role of Na^+^ in orthosteric D_2_R ligand binding, prompted us to investigate the role of Na^+^ in the action of SB269652. Here, we demonstrate that the action of SB269652 is exquisitely sensitive to the presence of Na^+^, so that in the absence of Na^+^, SB269652 no longer inhibits orthosteric ligand binding at the D_2_R. Using mutagenesis, MD simulations and derivatives of SB269652, we demonstrate that a Na^+^ ion, coordinated by Asp80^2.50^, is required for the high affinity binding of the THIQ PP. Interestingly, the particularly high Na^+^-sensitivity of SB269652, compared to both truncated fragments and structurally similar extended derivatives, is determined by the interaction between the 1*H*-indole-2-carboxamide moiety and the SBP.

## Results

### The binding of [^3^H]spiperone is insensitive to Na^+^

Saturation ligand binding studies were performed with the antagonist [^3^H]spiperone at wild-type (WT), D80^2.50^A and E95^2.65^A c-myc-D_2L_Rs stably expressed in membranes of Flp-In CHO cells. These assays were performed in the presence or absence of 100 mM NaCl. In the absence of NaCl, we included 100 mM of the organic cation N-methyl-D-glucamine (NMDG) to maintain buffer ionic strength^28,29^. Consistent with previous findings^12,28^, neither the affinity (*K*_d_) of [^3^H]spiperone nor the number of receptors labelled (B_max_) was significantly altered by the presence (100 mM NaCl, *K*_d_ = 0.04 nM, B_max_ = 237.0 fmol/mg) or absence of Na^+^ (100 mM NMDG, *K*_d_ = 0.05 nM, B_max_ = 216.1 fmol/mg) (Table 1, one-way ANOVA with Tukey’s post-hoc test, P_*K*d_ = 0.90, P_Bmax_ = 0.86). Our previous study revealed that Glu95^2.65^ within the SBP of the D_2_R was an important determinant of affinity and cooperativity of SB269652. Mutation of either Asp80^2.50^ or Glu95^2.65^ to alanine had no statistically significant effect on [^3^H]spiperone affinity (*K*_d_ = 0.1 nM and 0.06 nM, respectively, one-way ANOVA with Tukey’s post-hoc test, P_D80A_ = 0.06, P_E95A_ = 0.67) or number of receptors labelled (B_max_ = 215.4 fmol/mg and 225.5 fmol/mg, respectively, one-way ANOVA with Tukey’s post-hoc test, P_D80A = _0.86, P_E95A_ = 0.99) (Table 1). Furthermore, in the absence of Na^+^ neither the affinity nor the number of sites labelled were significantly altered at either the D80^2.50^A (*K*_d_ = 0.06 nM, B_max_ = 192.4 ± 10.7 fmol/mg, one-way ANOVA with Tukey’s post-hoc test, P_*K*d = _0.86, P_Bmax_ = 0.27) or E95^2.65^A (*K*_d_ = 0.08 nM, B_max_ = 196.6 fmol/mg, one-way ANOVA with Tukey’s post-hoc test, P_*K*d_ = 0.86, P_Bmax_ = 0.36) mutant.

**Table 1:** Binding parameters of orthosteric ligand and SB269652 derivatives at N-terminal c-Myc-tagged WT, D80^2.50^A, and E95^2.65^A D_2L_R constructs stably expressed in Flp-In CHO cell membranes.

		WT		D80^2.50^A		E95^2.65^A	
		100 mM NaCl	100 mM NMDG	100 mM NaCl	100 mM NMDG	100 mM NaCl	100 mM NMDG
**[** ^**3**^ **H]spiperone**	**p** ***K***_**d**_ **(** ***K***_**D**_ **nM)**	10.45 ± 0.06 (0.04)	10.31 ± 0.12 (0.05)	9.94 ± 0.11 (0.1)	10.24 ± 0.14 (0.06)	10.21 ± 0.09 (0.06)	10.10 ± 0.03 (0.08)
	**B**_**max**_ **fmol/mg**	237.0 ± 6.7	216.1 ± 10.8	215.4 ± 7.0	192.4 ± 10.7	225.5 ± 27.2	196.6 ± 8.8
**Dopamine**	**p*K***_**i**_ **(*K***_**i**_, **μM)**	5.59 ± 0.06 (2.6)	5.29 ± 0.16 (5.1)	5.58 ± 0.06 (2.6)	5.14 ± 0.16 (7.2)	5.5 ± 0.06 (3.1)	5.34 ± 0.09 (4.6)
**MIPS1071**	***pK***_**i**_ **(** ***K***_**i**_ **, μM)**	5.90 ± 0.07 (1.3)	4.92 ± 0.07* (12)	5.03 ± 0.07* (9.3)	5.14 ± 0.06* (7.2)	5.90 ± 0.09 (1.3)	5.12 ± 0.23* (7.6)
**MIPS1059**	**p** ***K***_**i**_ **(** ***K***_**i**_ **, μM)**	6.56 ± 0.06 (0.28)	5.76 ± 0.07* (1.7)	5.82 ± 0.07* (1.5)	5.83 ± 0.07* (1.5)	5.94 ± 0.07* (1.2)	5.32 ± 0.10*^#^ (4.8)
**SB269652**	**p** ***K***_**B**_^**a**^ **(** ***K***_**B**_ **, μM)**	6.45 ± 0.09 (0.35)	ND	ND	—	5.65 ± 0.10^ (2.2)	ND
	**logα** ^**b**^ **(α)**	−0.48 ± 0.08 (0.33)	ND	ND	—	−0.25 ± 0.06^ (0.56)	ND
**MIPS1726**	**p** ***K***_**B**_^**a**^ **(** ***K***_**B**_ **, nM)**	7.52 ± 0.05 (30.2)	6.40 ± 0.06* (398)	6.50 ± 0.06* (316)	—	6.91 ± 0.06* (123)	6.09 ± 0.08*^#¶^ (812)
	**logα** ^**b**^ **(α)**	−1.90 ± 0.08 (0.01)	−2.26 ± 0.39 (0.005)	−2.13 ± 0.27 (0.007)	—	−1.91 ± 0.12 (0.01)	−1.89 ± 0.31 (0.01)
**MIPS1868**	**p** ***K***_**B**_^**a**^ **(** ***K***_**B**_ **, nM)**	7.00 ± 0.07 (100)	6.20 ± 0.08* (631)	6.20 ± 0.10* (631)	6.33 ± 0.09* (468)	6.67 ± 0.03* (212)	6.21 ± 0.12*^#^ (617)
	**logα** ^**b**^ **(α)**	−1.45 ± 0.12 (0.04)	−1.48 ± 0.12 (0.03)	−0.62 ± 0.06* (0.23)	−0.87 ± 0.12* (0.13)	−1.39 ± 0.11 (0.04)	−1.24 ± 0.16 (0.06)

Data is the mean ± SEM of three individual experiments. ^a^Estimate of the negative logarithm of the equilibrium dissociation constant. ^b^Estimate of the logarithm of the cooperativity factor between the modulator and [^3^H]spiperone. ^c^experiments perfromed using 0.5 nM [^3^H]spiperone. ^*^Statistically different from corresponding affinity estimate of ligand at WT D_2L_R in the presence of 100 mM NaCl (p < 0.05, one-way ANOVA, Tukey’s post hoc test). ^^^Statistically different from corresponding affinity estimate of ligand at WT D_2L_R in the presence of 100 mM NaCl (p < 0.05, unpaired, two-tailed Student’s t-test). ^#^Statistically different from corresponding affinity estimate of ligand at E95^2.65^A in the presence of 100 mM NaCl (p < 0.05, one-way ANOVA, Tukey’s post hoc test). ^¶^Statistically different from corresponding affinity estimate of ligand at WT in the presence of 100 mM NMDG (p < 0.05, one-way ANOVA, Tukey’s post hoc test). ND signifies that no inhibition of [^3^H]spiperone binding was detected. N/A signifies that the parameter was not calculated. ‘-‘ signifies that no binding experiments were performed for that condition.

**Figure 1:**
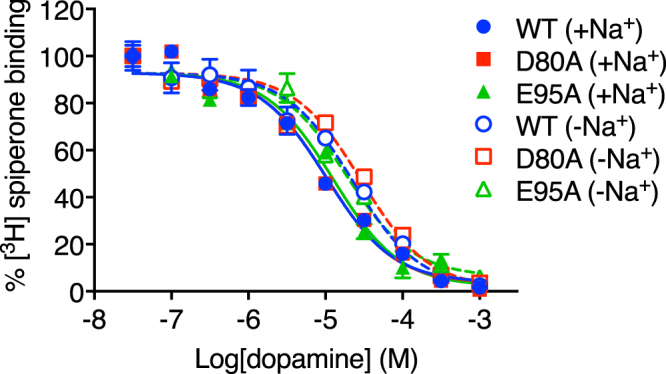
The affinity of dopamine is not significantly changed in the absence of Na^+^. Competition binding experiments performed using membranes of FlpIn CHO cells stably expressing a human Myc-hD_2L_R and the antagonist [^3^H]spiperone reveal that in the presence of 100 mM NaCl (closed symbols) the mutations D80^2.50^A and E95^2.65^A do not significantly change the affinity of dopamine relative to that at the wild type (WT) receptor. In the absence of Na^+^ (open symbols), whereby 100 mM of the organic cation N-methyl-D-glucamine (NMDG) is included in the buffer to maintain ionic strength, no change in dopamine affinity is observed at the WT, D80^2.50^A or E95^2.65^A hD_2L_Rs relative to the affinity determined in the presence of Na^+^.

The insensitivity of [^3^H]spiperone binding to the presence of Na^+^ makes this ligand an excellent probe radioligand with which to investigate Na^+^ sensitivity in the action of other D_2_R ligands. Consistent with published results^12,22^, in the presence of 100 μM GppNHp, the affinity of the endogenous agonist dopamine (100 mM NaCl, *K*_i_ = 2.6 μM) was not significantly altered in the absence of Na^+^ (100 mM NMDG, *K*_i_ = 5.1 μM, one-way ANOVA with Tukey’s post-hoc test, P = 0.41) or upon mutation of Asp80^2.50^ to alanine (*K*_i_ = 2.8 μM, one-way ANOVA with Tukey’s post-hoc test, P > 0.99) (Fig. 1, Table 1). Mutation of E95^2.65^ to alanine did not significantly alter dopamine affinity either (*K*_i_ = 3.1 μM, one-way ANOVA with Tukey’s post-hoc test, P = 0.98) (Table 1).

### SB269652 displays no activity in the absence of Na^+^

**Figure 2:**
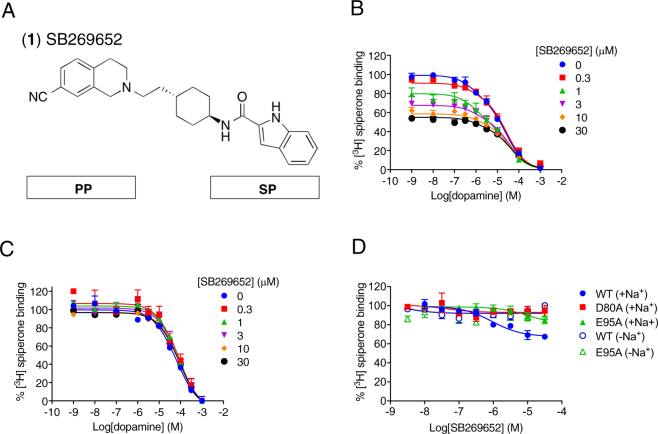
The NAM SB269652 has no effect at the hD_2L_R in the absence of Na^+^. (**A**) SB269652 (**1**) is comprised of a 7-cyano-tetrahydroisoquinoline primary pharmacophore and a indole-2-carboxamide secondary pharmacophore (SP) linked by a cyclohexylene spacer. (**B**) In the presence of 100 mM NaCl, SB269652 acts to inhibit the binding of both dopamine and [^3^H]spiperone in a competition binding assay using membranes from FlpIN CHO cells stably expressing the WT hD_2L_R. These data could be fitted to an allosteric ternary complex model to derive a value of affinity for SB269652 and values of cooperativity with both dopamine and [^3^H]spiperone (Table 1). (**C**) In the absence of sodium ions, but the presence of 100 mM NMDG, no effect of SB269652 upon dopamine or [^3^H]spiperone affinity at the hD_2L_R is observed. (**D**) In competition binding experiments performed using membranes of FlpIn CHO cells stably expressing a human Myc-hD_2L_R and the antagonist [^3^H]spiperone reveal that in the presence of 100 mM sodium (closed symbols) SB269652 acts to partially displace the radioligand consistent with the action of a negative allosteric modulator (NAM). SB269652 has no effect in the absence of Na^+^ (open symbols) or at the D80^2.50^A and E95^2.65^A receptor mutants.

In the presence of 100 mM NaCl, increasing concentrations of SB269652 caused both an incomplete displacement of [^3^H]spiperone and modulation of dopamine affinity (Fig. 2B). These data were fit to an allosteric ternary complex model to obtain a value of affinity for SB269652 (*K*_B_ = 355 nM) and cooperativity with dopamine (logα = −0.54 ± 0.02, α = 0.29) and [^3^H]spiperone (logα’ = −0.45 ± 0.08, α’ = 0.35, Fig. 2B). In contrast, in the absence of Na^+^, SB269652 failed to modulate dopamine affinity or displace [^3^H]spiperone binding (Fig. 2C, Table 1). Thus, we were unable to detect any evidence of SB269652 binding in the absence of Na^+^. We extended this study to determine whether this exquisite Na^+^ sensitivity reflects a need for a Na^+^ ion bound within the conserved Na^+^-binding pocket by measuring the ability of SB269652 to displace [^3^H]spiperone binding to the D80^2.50^A mutant (Fig. 2D, Table 1), in which the conserved Na^+^ site is disrupted. Similar to our observations in the absence of Na^+^, SB269652 no longer inhibited the binding of [^3^H]spiperone at this mutant even in the presence of 100 mM NaCl. Thus, SB269652 binding is dependent on the presence of a Na^+^ ion in the conserved Na^+^-binding pocket coordinated by Asp80^2.50^.

### A Na^+^ ion coordinated by Asp80^2.50^ is important for high affinity binding of the THIQ moiety within the OBS

**Figure 3:**
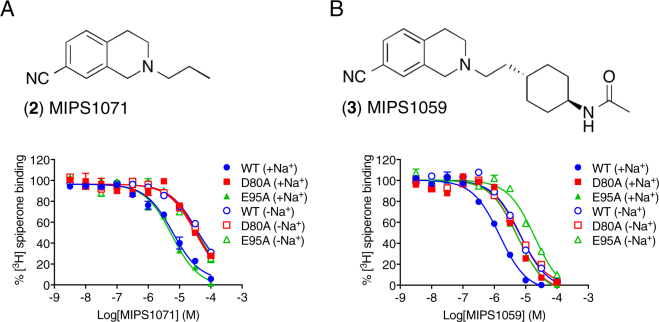
Fragments of SB269652 contaiing the THIQ core display competitve pharmacology and lower affinity in the absence of an Na^+^ ion coordinated by D80^2.50^. (**A**) In the presence of 100 mM NaCl (closed symbols) the fragment of SB269652, 2-propyl-1,2,3,4-tetrahydroisoquinoline-7-carbonitrile (2, MIPS1071) acts to completely displace [^3^H]spiperone in a competition binding assay using membranes from FlpIN CHO cells stably expressing the WT hD_2L_R. The affinity of MIPS1071 is unchanged at the E95^2.65^A receptor mutant but decreased at the D80^2.50^A receptor mutant. In the absence of 100 mM NaCl (100 mM NMDG, open symbols), the affinity of MIPS1071 is decreased at the WT D_2L_R and E95^2.65^A mutant but unchanged at the D80^2.50^A mutant. (**B**) In the presence of 100 mM NaCl (closed symbols) *N*-((*trans*)-4-(2-(7-cyano-3,4- dihydroisoquinolin-2(1 *H*)yl)ethyl)cyclohexyl)acetamide (3, MIPS1059), a fragment that includes the carboxamide but not the indole moiety of SB269652, acts to completely displace [^3^H]spiperone in a competition binding assay using membranes from FlpIN CHO cells stably expressing the WT hD_2L_R. The affinity of MIPS1059 is decreased at both the E95^2.65^A and D80^2.50^A receptor mutants. In the absence of 100 mM NaCl (100 mM NMDG, open symbols), the affinity of MIPS1071 is significantly decreased at the WT D_2L_R and E95^2.65^A mutant but unchanged at the D80^2.50^A mutant.

Our previous studies have shown that the presence of a Na^+^ in the conserved Na^+^-binding pocket can modulate the affinity of some but not all D_2_R antagonists^24^. With the hypothesis that the THIQ PP of SB269652 requires Na^+^ bound in the conserved pocket to bind with high affinity to the OBS, we performed competition binding experiments with two orthosteric fragments of SB269652; 2-propyl-1,2,3,4-tetrahydroisoquinoline-7-carbonitrile (**2**, MIPS1071) and a more extended fragment *N*-((trans)-4-(2-(7-cyano-3,4-dihydroisoquinolin-2(1*H*)yl)ethyl)cyclohexyl)acetamide (**3**, MIPS1059). Both MIPS1071 and MIPS1059 have previously been shown to behave as competitive antagonists of dopamine in functional assays and acted to completely displace the binding of [^3^H]spiperone (*K*_i_ = 1.3 μM and 275 nM respectively, Fig. 3)^2^. MIPS1071 displayed a significant loss of affinity both in the absence of Na^+^ and at the D80^2.50^A mutant receptor (Fig. 3A, Table 1). In both cases, MIPS1071 was unable to completely displace the binding of [^3^H]spiperone at a concentration of 100 mM. However, by assuming complete displacement, we could estimate values of affinity in the Na^+^-free condition (p*K*_i_ = 4.92 ± 0.07, *K*_i_ = 12 μM) and at the D80^2.50^A mutant receptor (p*K*_i_ = 5.03 ± 0.07, *K*_i_ = 9.3 μM) that correspond to a 9-fold and 7-fold loss in affinity respectively (one-way ANOVA, Tukey’s post-hoc test, P_−Na+_ < 0.0001, P_D80A_ < 0.0001). Similarly, MIPS1059 displayed a 6-fold or 5-fold decrease in affinity in the absence of Na^+^ (*K*_i_ = 1.7 μM,) or at the D80^2.50^A mutant (*K*_i_ = 1.5 μM) respectively (Fig. 3B, Table 1, one-way ANOVA, Tukey’s post-hoc test, P_−Na+_ < 0.0001, P_D80A_ < 0.0001). In both cases, no additional effect of the Na^+^-free condition was observed at the D80^2.50^A mutant. Thus, the Na^+^ sensitivity of both orthosteric fragments provides evidence that a Na^+^ ion, coordinated by Asp80^2.50^, is required for the high affinity binding of the THIQ PP of SB269652 within the OBS. However, given that in the absence of Na^+^ or at the D80^2.50^A mutant these orthosteric fragments display a maximal 10-fold decrease in affinity, if SB269652 displayed a similar loss of affinity then we would still be readily able to detect the activity of SB269652 in our binding studies. Thus, the absence of Na^+^ must either have a greater effect upon SB269652 affinity and/or an additional effect upon SB269652 cooperativity. The lack of effect of SB269652 in the absence of Na^+^ means that it is not possible to distinguish these two possibilities. However, SB269652 displays relatively modest affinity for the D_2_R and weak negative cooperativity, resulting in only a small window to detect changes in pharmacology. To address this, we extended our study to extended derivatives of SB269652 that display higher affinity and negative cooperativity with dopamine.

### A Na^+^ ion coordinated by Asp80^2.50^ modulates the affinity and cooperativity of high affinity derivatives of SB269652

**Figure 4:**
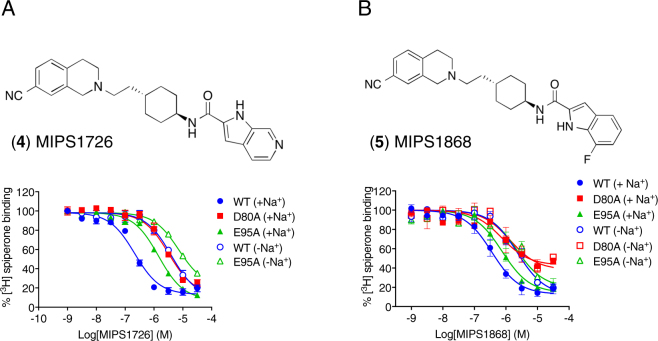
High affinity Derivatives of SB269652 display lower affinity and cooperatiivty in the absence of a sodium ion coordinated by D80^2.50^. (**A**) In the presence of 100 mM NaCl (closed symbols) the azaindole derivative of SB269652, (4, MIPS1726) acted to partially displace 0.5 nM [^3^H]spiperone in a competition binding assay using membranes from FlpIN CHO cells stably expressing the WT hD_2L_R. These data could be fit to an allosteric ternary complex model to derive a value of affinity and cooperativity with [^3^H]spiperone (Table 1). In the presence of Na^+^, MIPS1726 displayed lower affinity at the E95^2.65^A and D80^2.50^ receptor mutants (Table 1). In the absence of Na^+^ (100 mM NMDG, open symbols) MIPS1726 displayed lower affinity at the WT and E95^2.65^A D_2L_Rs but no change in affinity at the D80^2.50^A mutant (Table 1). (**B**) In the presence of 100 mM NaCl (closed symbols) the 7-fluoro-indole-2-carboxamide derivative of SB269652 (5, MIPS1868) acted to partially displace 0.15 nM [^3^H]spiperone in a competition binding assay using membranes from FlpIN CHO cells stably expressing the WT hD_2L_R. These data could be fit to an allosteric ternary complex model to derive a value of affinity for SB269652 and cooperativity with [^3^H]spiperone (Table 1). In the presence of Na^+^, MIPS1868 displayed lower affinity at the E95^2.65^A receptor mutant but lower affinity and cooperativity at the D80^2.50^ receptor mutant (Table 1). In the absence of Na^+^ (100 mM NMDG, open symbols) MIPS1868 displayed lower affinity at the WT D_2L_R but no change in affinity at the E95^2.65^A or D80^2.50^A mutants.

To understand the effect of Na^+^ on the affinity and/or cooperativity of an extended allosteric compound, we used an azaindole derivative (**4**, MIPS1726) that differs through the incorporation of an additional nitrogen at the 6-position of the indole-2-carboxamide SP of SB269652. In our competition binding experiments using 0.15 nM [^3^H]spiperone, increasing concentrations of MIPS1726 completely displaced the radioligand (p*K*_B_ = 8.01 ± 0.05) most likely reflecting high negative cooperativity. Consistent with such a mechanism, in the presence of a higher concentration of [^3^H]spiperone (0.5 nM, Fig. 4A), MIPS1726 acted to partially displace the radioligand consistent with an allosteric mode of action. These data could be fit to an allosteric ternary complex model to estimate a value of affinity (*K*_B_ = 30 nM) and negative cooperativity with [^3^H]spiperone (α = 0.01, Table 1). Unlike the complete loss of detectable action of SB269652 in the absence of Na^+^ or at the D80^2.50^A mutant receptor, MIPS1726 retained NAM activity but displayed a significant >10-fold loss in affinity (*K*_B_ = 398 nM and 316 nM, respectively, one-way ANOVA with Tukey’s post-hoc test P_−Na+_ < 0.0001, P_D80A_ < 0.0001, Table 1) with no change in negative cooperativity. Therefore, the absence of a Na^+^ within the Na^+^-binding pocket, confers a loss of affinity for this extended allosteric compound but does not affect cooperativity.

We then extended our investigation to another higher-affinity allosteric derivative of SB269652, MIPS1868 (**5**), that differs from SB269652 through the substitution of a fluorine at the 7-position of the indole-2-carboxamide SP (Fig. 4B). This derivative also displays greater negative cooperativity with [^3^H]spiperone as compared to SB269652 (*K*_B_ = 100 nM, α = 0.04, Table 1). Interestingly, we observed different effects of the Na^+^-free condition compared to the mutation of Asp80^2.50^ on this derivative. At the WT D_2_R, MIPS1868 displayed a modest 6-fold loss of affinity (one-way ANOVA with Tukey’s post-hoc test, P < 0.002) in the absence of Na^+^ but no significant change in negative cooperativity (one-way ANOVA with Tukey’s post-hoc test, P > 0.99). In contrast, the D80^2.50^A mutation caused a similar 6-fold loss in affinity (one-way ANOVA with Tukey’s post-hoc test, P < 0.002) plus a 4-fold loss of negative cooperativity (one-way ANOVA with Tukey’s post-hoc test, P < 0.002). Thus, for MIPS1868, this mutation has an additional effect upon cooperativity that cannot be reconciled solely through the role of this residue to coordinate a Na^+^ ion in the Na^+^-binding pocket. It should be noted that both MIPS1726 and MIPS1868 displayed relatively modest losses of affinity (10-fold and 6-fold) in the absence of Na^+^ as compared to the complete lack of activity of SB269652. Therefore SB269652 must display a much greater loss of affinity or cooperativity to explain its lack of activity at the D_2_R in this condition. Indeed, for SB269652 to display no activity up to 30 μM then the absence of Na^+^ must confer a greater than 80-fold loss of affinity.

### The absence of Na^+^ disrupts the binding modes of SB269652 and its derivatives

Our previous modelling studies predicted that SB269652 binds to the D_2_R in an extended pose in which the THIQ PP occupies the OBS while the 1*H*-indole-2-carboxamide SP of SB269652 extends away from the OBS to interact with residues within the SBP^2^. In particular, these studies suggested that a hydrogen bond interaction between Glu95^2.65^ and the indolic NH is important for the affinity and cooperativity of SB269652. To test how the absence of the Na^+^ bound at the canonical Na^+^-binding site near Asp80^2.50^ would alter the binding poses of SB269652 and its aforementioned derivatives, we carried out comparative MD simulations of SB269652, MIPS1059, MIPS1726, and MIPS1868 bound to the D_2_R. For all these complexes, we simulated both the Na^+^-bound and Na^+^-unbound conditions (See Methods and Table 2).

**Table 2:** Summary of MD simulations of D_2_R in complex with SB269652 and its derivatives.

Ligand	Na^+^	Number of trajectories	Total Length (µs)
**SB269652**	+	37	18.0
	−	4	9.0
**MIPS1059**	+	2	4.8
	−	2	4.8
**MIPS1726**	+	2	4.2
	−	4	6.6
**MIPS1868**	+	4	6.0
	−	2	4.8
**Total**		**57**	**58.2**

**Figure 5:**
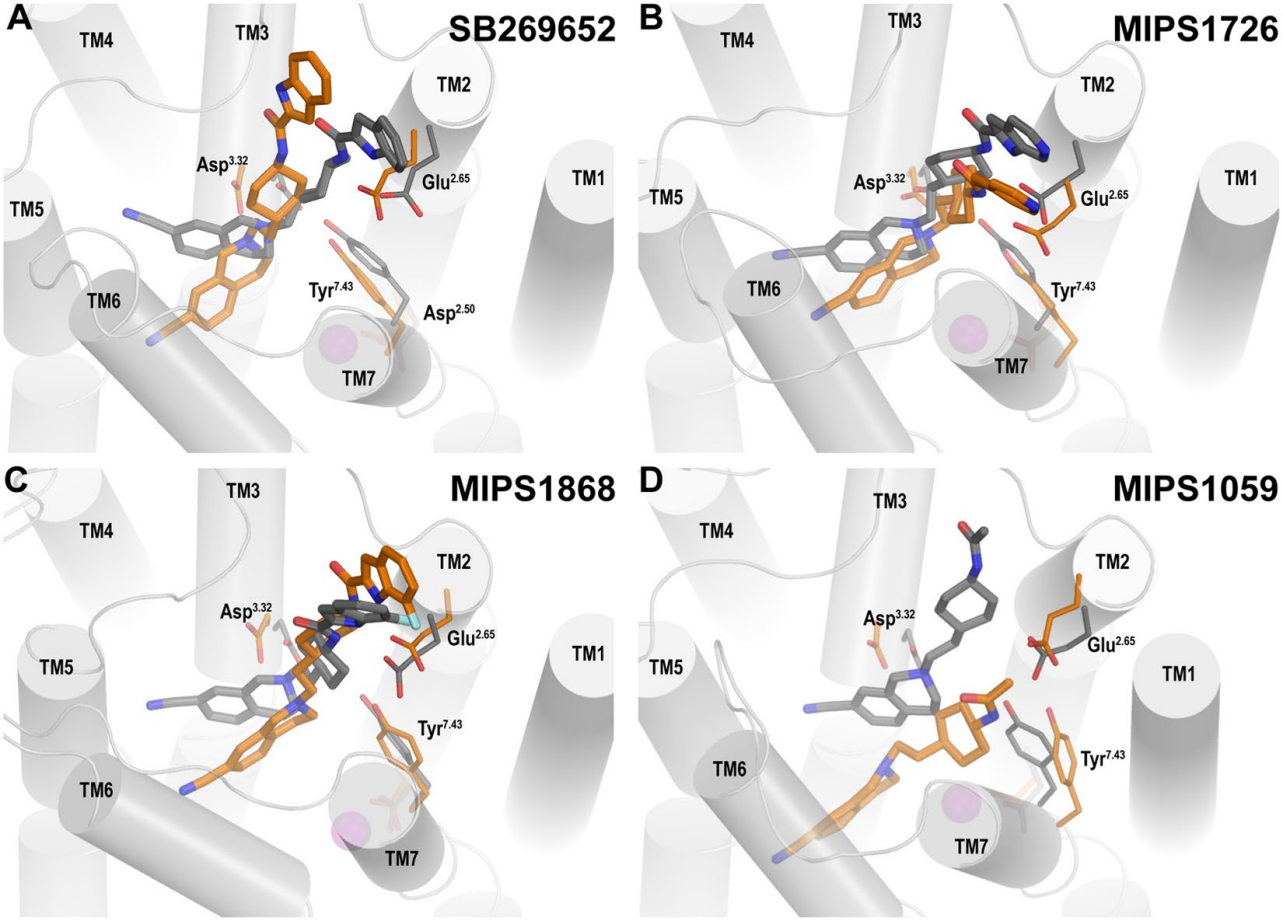
The absence of Na^+^ disrupts the binding modes of SB269652 and its derivatives at D_2_R. The binding modes of ligands at the D_2_R in the presence (grey) and absence (orange) of the Na^+^ bound near Asp80^2.50^ show significant differences. The absence of bound Na^+^ significantly weakens the ionic interaction between the Asp114^3.32^ and the positively charged N1 atom of SB269652 and its derivatives. The THIQ moiety of these ligands shows a common tendency to move towards the TM5-TM6 interface and deeper into the OBS. In the absence of Na^+^, high-affinity ligands (MIPS1726 and MIPS1868) form strong hydrogen bonds with Glu95^2.65^ (Supplementary Figure 2), in contrast to SB269652.

For all these compounds, whereas in the Na^+^-bound conditions the THIQ moiety consistently faces the TM4-TM5 interface in the OBS, in the Na^+^-unbound conditions this moiety has a strong tendency to move towards the TM5-TM6 interface and deeper into the OBS (Fig. 5A–D, Supplementary Figures 1 and 2). In addition, the salt-bridge interaction between the Asp114^3.32^ and the protonated amine nitrogen (N1, see Supplementary Figure 1) of the ligands is significantly weakened compared to that in the Na^+^-bound conditions (Supplementary Figures 1 and 2). We hypothesised that these changes are responsible for the observed differences in the binding affinities of these Na^+^-sensitive ligands. Indeed, the results of our MM/GBSA calculations of the ligand binding energies indicate that SB269652 and its derivatives bind more favourably in the Na^+^-bound conditions than their corresponding Na^+^-unbound conditions (Supplementary Figure 3). We were also interested in investigating how the Na^+^-unbound condition would affect the orientation of the SP of SB269652 and derivatives within the SBP. We found that the interactions between Glu95^2.65^ and the amide nitrogen (N3) or the nitrogen of the aryl moiety (N4) of MIPS1868 and MIPS1726 were consistently maintained in the Na^+^-unbound condition; in comparison, the corresponding interactions were predicted to be much weaker for SB269652 (Supplementary Figure 2). This suggests that the modifications to the SP of MIPS1726 and MIPS1868 contribute additional interactions within the SBP that confer the higher affinity of these ligands. This in turn may explain why the Na^+^-unbound condition ablates the action of SB269652, whereas the NAM activities of MIPS1726 and MIPS1868 are still detectable in the absence of Na^+^.

### Na^+^-binding modulates the interaction of SB269652 derivatives with the SBP

We extended our study to further explore how Na^+^ binding might influence the interaction between the indolic NH of SB269652 and the SBP residue Glu95^2.65^. Consistent with our previous findings, the affinity of SB269652 (6-fold, Student’s two-tailed unpaired t-test, P = 0.004) was decreased at the E95^2.65^A mutant^2^. As observed at the WT D_2_R, in the absence of Na^+^, SB269652 was unable to inhibit the binding of [^3^H]spiperone at the E95^2.65^A mutant (Fig. 2C) - thus we could not measure an additional effect of the Na^+^-free condition. The affinity of the orthosteric fragment MIPS1071 was not changed, as compared to WT, by the mutation E95^2.65^A in the presence or absence of Na^+^ (Table 1, one-way ANOVA with Tukey’s post-hoc test, P_+Na+_ > 0.99, P_−Na+_ = 0.55). However, the extended fragment MIPS1059 displayed a significant 5-fold affinity reduction at the E95^2.65^A mutant compared to the WT in the presence of Na^+^ (Table 1, one-way ANOVA with Tukey’s post-hoc test, P = 0.0002). This loss of affinity may reflect the loss of a hydrogen bond interaction between Glu95^2.65^ and the carboxamide NH of MIPS1059. A significant 3-fold loss of affinity at the E95^2.65^A mutant was also observed in the absence of Na^+^ (one-way ANOVA with Tukey’s post-hoc test, P = 0.01). Similar to observations at the WT D_2_R, MIPS1059 displays 5-fold lower affinity at the E95^2.65^A mutant in the absence of Na^+^ compared to the E95^2.65^A mutant in the presence of Na^+^ (one-way ANOVA with Tukeys’s post-hoc test, P = 0.0008).

The extended SB269652 derivative, MIPS1726, displayed a significant 4-fold loss in affinity at the E95^2.65^A (*K*_B_ = 123 nM) mutant compared to WT D_2_R (one-way ANOVA with Tukey’s post-hoc test, P = 0.0003). The absence of Na^+^ caused a further significant 7-fold decrease in affinity at the E95^2.65^A mutant consistent with these effects being additive, similar to our observations with the fragment MIPS1059 (one-way ANOVA with Tukey’s post-hoc test, P < 0.0001). The loss of affinity for MIPS1726 at the E95^2.65^A mutant as compared to the WT D_2_R in both the presence and absence of Na^+^ is consistent with presence of a hydrogen bond interaction between the azaindole moiety of MIPS1726 and Glu95^2.65^ in both conditions (Table 1, Fig. 4B). The mutation E95^2.65^A caused a modest 2-fold decrease in affinity of the extended SB269652-derivative (one-way ANOVA, Tukey’s post-hoc test, P = 0.049), MIPS1868, in the presence of Na^+^. The Na^+^-free condition caused a further 3-fold decrease in the affinity of MIPS1868 at this mutant (one-way ANOVA, Tukey’s post-hoc test, P = 0.02). However, in contrast to the 2-fold difference observed for MIPS1726, comparison of the affinity of MIPS1868 at the WT D_2_R and the E95^2.65^A mutant in the absence of Na^+^ revealed no significant difference (one-way ANOVA with Tukey’s post-hoc test, P > 0.99). Interestingly, results from our MD simulations revealed that the extra nitrogen (N5 atom) or the fluoro atom on the SP of MIPS1726 and MIPS1868, respectively, form interactions with the hydroxyl group of Tyr34^1.32^ in Na^+^-bound condition (Supplementary Figure 4). In the absence of Na^+^, the interaction between the fluoro atom of MIPS1868 and Tyr34^1.32^ remained stable, whereas the interaction between the N5 atom of MIPS1726 and Tyr34^1.32^ weakens, which suggests that interaction between MIPS1868 and Tyr34^1.32^ is relatively strong and may mask the presence and absence of the interactions with Glu95^2.65^.

Finally, it is interesting to note that the observed difference in affinity of MIPS1059, MIPS1726 and MIPS1868 between the WT and E95^2.65^A mutant was significantly and consistently (2-fold) greater in the presence of Na^+^ as compared to the difference observed in the absence of Na^+^ (Supplementary Table 1). This suggests that in all cases the presence of Na^+^ has a subtle but significant effect upon modulating the interaction between these SB269652 derivatives and the SBP.

## Discussion

The potentially conserved functional role of Na^+^ at GPCRs^30^ prompted us to explore the impact of Na^+^ binding on the pharmacology of SB269652, the first small-molecule NAM identified at the D_2_R. We found that SB269652 displayed exquisite sensitivity to Na^+^, losing its modulatory effect in the absence of this ion. Recent crystallographic, computational modelling and biochemical efforts have revealed a Na^+^ binding site beneath the OBS that includes a highly conserved aspartate (Asp^2.50^)^15,16^, which allowed us to carry out a mechanistic study using both mutagenesis and computational approaches.

A recent study demonstrated that a μ opioid receptor PAM, BMS986122, decreased the ability of Na^+^ ions to inhibit agonist binding. This observation can be accommodated within a two-state model, whereby BMS986122 favours an active receptor conformation with a collapsed Na^+^-binding pocket^27^. The action of SB269652 cannot be reconciled within such a model because it exerts negative allosteric modulation with respect to both agonist and antagonist binding. Previous studies of the D_2_R revealed that in the absence of Na^+^, the competitive antagonist raclopride exerted non-competitive effects upon both its own binding and that of the antagonist [^3^H]spiperone^28,31^. These observations were rationalised within a model of a D_2_R dimer whereby the binding of one molecule of raclopride to one protomer of this dimer, exerted negative cooperativity in the binding of another molecule to the orthosteric site of the other protomer^28^. We have previously proposed a novel mechanism of action for SB269652, where it binds as a bitopic ligand at one protomer and exerts negative cooperativity for the binding and action of dopamine at an adjacent protomer^2^. However, SB269652 and its derivatives MIPS1726 and MIPS1868 all display negative allosteric pharmacology with [^3^H]spiperone in the presence of Na^+^ ions. Thus, the non-competitive action of these ligands cannot be explained by the mechanism proposed to explain the behaviour of [^3^H]raclopride in the absence of Na^+^^28^.

Some classes of D_2_R antagonists such as substituted benzamides show a significant loss of affinity in the Na^+^-unbound state^12^. We previously demonstrated that in the Na^+^-unbound state the weakening of a critical hydrogen bond interaction between the Asp^3.32^ and Tyr^7.43^ residues, positioned above the Na^+^-binding site, led to non-optimal binding modes for these Na^+^-sensitive antagonists^24^. Results from these MD simulations were consistent with results from a previous study that demonstrated that mutation of Tyr^7.43^ to cysteine dramatically decreased the affinity of sulpiride (185-fold), while only a subtle loss of affinity was observed for the Na^+^-insensitive N-methyl-spiperone (3-fold)^32^. Ligands that displayed Na^+^-insensitivity, such as spiperone, make additional contacts within the ligand binding pocket that mask the impact of the absence of bound Na^+^ ^24^.

The results of mutagenesis and MD simulations in this study reveal that the Na^+^ sensitivity of SB269652 can be reconciled with a mechanism whereby the occupancy of the OBS by the THIQ moiety is dependent upon Na^+^. Indeed, the two fragments of SB269652 (MIPS1071 and MIPS1059) that contain the THIQ moiety and act as competitive antagonists displayed pronounced Na^+^ sensitivity, akin to that of the substituted benzamides^2,23,24^. This Na^+^ sensitivity is mediated by the interaction of Na^+^ with Asp80^2.50^, as mutation of this residue to alanine produced an identical effect in either the presence or absence of Na^+^. Consistent with this hypothesis, the predominant effect of the Na^+^-free condition was a decrease in affinity of the high affinity allosteric SB269652 analogues MIPS1726 and MIPS1868.

However, while the lack of effect of SB269652 in the absence of Na^+^ may reflect a significant decrease in affinity for the OBS, the absence of a Na^+^ coordinated by Asp80^2.50^ caused a relatively modest decrease in affinity of the THIQ-containing fragments MIPS1071 and MIPS1059 (10-fold and 6-fold, respectively). Similarly, the higher affinity SB269652 derivatives MIPS1726 and MIPS1868 show 12-fold and 5-fold lower affinities respectively in the absence of Na^+^. In comparison, the lack of activity of SB269652 in the absence of Na^+^ up to a concentration of 30 μM requires a much larger (greater than 80-fold) decrease in affinity. This suggests that the absence of Na^+^ exerts effects of greater magnitude upon SB269652 affinity and/or additional effects such as a decrease in negative cooperativity. In agreement with a larger impact upon SB269652 affinity, our MD simulations showed that the interactions of Glu95^2.65^ in the SBP with the protonated amide nitrogen and the protonated nitrogen of the aryl moiety of MIPS1868 and MIPS1726 were maintained even in the Na^+^-free condition, whereas the corresponding interactions were predicted to be much weaker for SB269652. This suggests that the modifications to the SP of MIPS1726 and MIPS1868 relative to the 1*H*-indole-2-carboxamide SP of SB269652 contribute additional interactions within the SBP that confer the higher affinities of these two derivatives and may explain why the Na^+^-free condition ablates the action of SB269652, while retaining detectable NAM activities of MIPS1726 and MIPS1868. The orthosteric fragments MIPS1071 and MIPS1059 lack this SP. Interestingly, the addition of the indole moiety of SB269652 does not confer a gain in affinity relative to MIPS1059. Therefore, one possible explanation for the greater effect of the Na^+^-free condition upon SB269652 as compared to these orthosteric fragments, is that the addition of the SP of SB269652 not only confers a relatively weak interaction with Glu95^2.65^ but decreases the strength of the interaction with residues within the OBS, together conferring a greater sensitivity to Na^+^.

It is interesting to note that the observed difference in affinity of MIPS1059, MIPS1726 and MIPS1868 between the WT and E95^2.65^A mutant was significantly and consistently (2-fold) greater in the presence of Na^+^ as compared to the difference observed in the absence of Na^+^ (Supplementary Table 1). Such a difference may reflect a change in orientation of these ligands such that the interaction with Glu95^2.65^ has less impact upon ligand affinity, or that Na^+^ within the conserved Na^+^-binding pocket may modulate the shape of the SBP. Indeed, the presence of Na^+^ within this conserved binding pocket was proposed to modulate the affinity of 1,4-disubstituted piperidines/piperazines for the D_2_R by increasing the accessibility of the aryl substituents with V91^2.61^, a residue within the SBP^25^. However, these data may also be consistent with a transient Na^+^ binding interaction with E95^2.65^ proposed by Selent and coworkers that may in turn modulate the interaction of extended compounds such as SB269652 with the SBP^26^.

Finally, the D80^2.50^A mutation caused a similar decrease in the affinity of MIPS1726 as observed in the Na^+^-free condition and no change in negative cooperativity was observed. In contrast this mutation caused an additional 6-fold decrease in the negative cooperativity of MIPS1868, an effect not observed at the WT D_2_R in the Na^+^-free condition. This suggests that this residue plays a role in determining the cooperativity of MIPS1868 that cannot be reconciled simply through its role in coordinating a Na^+^ ion. These differential effects appear to depend on the structure of the aryl amide tail moiety, which differs between MIPS1726 and MIPS1868. Thus, the interaction between the aryl amide tail moiety of SB269652 or its derivatives and the SBP is important in determining not only affinity and the magnitude of negative cooperativity but also the effect of Na^+^-binding and the D80^2.50^A mutation^2^. We speculate that the absence of the Na^+^ coordinated by Asp80^2.50^ would alter the position of the orthosteric THIQ moiety bound in the OBS, leading to a change in position of the aryl amide moiety within the SBS. It is interesting to note that in the absence of Na^+^, the mutation E95^2.65^A has no additional effect upon the affinity of MIPS1868 but caused a 2-fold decrease in the affinity of MIPS1726, an effect that may result from a distinct orientation of these two ligands within the SBS in the Na^+^-free condition. Indeed the resulting poses of these two ligands from our MD simulations show noticeable differences in the SBS, with the indole ring of MIPS1868 being positioned more extracellularly than that of MIPS1726 in the Na^+^-free condition, when they lose the interaction with Asp^3.32^ (Supplementary Figure 4). Furthermore, the interaction between the N5 atom of MIPS1726 and Tyr34^1.32^ was weakened in the absence of Na^+^ whereas the interaction of the same residue with fluoro atom of MIPS1868 and Tyr34^1.32^ remained stable and may mask the effect of the E95^2.65^A mutation.

To date, investigations into the influence of Na^+^ upon dopamine receptor function have been limited to effects on the affinity and efficacy of competitive orthosteric ligands. Here we reveal profound Na^+^-sensitivity for the action of the NAM, SB269652. This effect is predominantly mediated by a Na^+^ ion located within the conserved Na^+^-binding site and coordinated by Asp80^2.50^. Our previous studies have proposed a mechanism of action for SB269652, whereby it adopts a dual orthosteric/allosteric (bitopic) mode of action at the D_2_R. This study reveals that the bound Na^+^ is required for the high affinity binding of the THIQ moiety of SB269652 and its analogues within the OBS. These observations are consistent with a role for the Na^+^ in shaping the OBS of the D_2_R, and in particular to orient Asp114^3.32^ in forming a salt bridge with the protonated tertiary amine of the THIQ moiety as has been observed for some, but not all, classes of competitive D_2_R antagonists. Our study also suggests that the complete loss of detectable action of SB269652 in the absence of Na^+^ reflects a difference in interactions within the SBP as compared to structurally similar derivatives. Thus, by revealing a synergistic interaction between Na^+^ and SB269652 to modulate orthosteric ligand binding at the D_2_R we provide further insight into the binding mode and mechanism of this new class of allosteric modulator. This can form the basis for fine-tuning such modulatory effects in future allosteric drug design efforts at this receptor.

## Methods

### Materials

Dulbecco’s modified Eagle’s medium, Flp-In CHO cells, and hygromycin B were purchased from Invitrogen (Carlsbad, CA). Fetal bovine serum (FBS) was purchased from ThermoTrace (Melbourne, VIC, Australia). [^3^H]spiperone and Ultima Gold scintillation cocktail were from PerkinElmer (Boston, MA). All other reagents were purchased from Sigma-Aldrich (St. Louis, MO). All compounds were synthesized as described previously^2,3^ with the exception of MIPS1868 and MIPS1726, which were synthesised as described in supplementary information.

### Molecular biology

cDNA in pcDNA3.1+ encoding the long isoform of the wild-type human D_2_ dopamine receptor (D_2L_R) was obtained from Missouri University of Science and Technology (http://www.cdna.org/). Oligonucleotides were purchased from GeneWorks (Hindmarsh, Australia). An N-terminal c-Myc epitope tag (EQKLISEEDL) was introduced to the sequence of the D_2L_R, and flanking AttB sites were introduced to the WT D_2L_R by overlap extension PCR to allow subcloning into the pDONR201TM vector. The c-Myc–tagged WT D_2L_R receptor construct in pDONR201TM was transferred into the pEF5/frt/V5/dest vector using the LR clonase enzyme mix (Invitrogen). Desired mutations were introduced using the Quikchange site-directed mutagenesis kit (Agilent). Mutations were confirmed by DNA sequencing (Australian Genome Research Facility, Melbourne, Australia). Receptor constructs in pEF5/frt/V5/dest were used to transfect Flp-In CHO cells.

### Cell culture and membrane preparation

Flp-In CHO cells (Invitrogen) were transfected with the pOG44 vector encoding Flp recombinase and the pDEST vector encoding the wild-type or mutant c-Myc-D_2L_R at a ratio of 9:1 using polyethylenimine (Polysciences, Warrington, PA) as transfection reagent^2^. 24 hours after transfection the cells were subcultured and the medium was supplemented with 700 μg/ml hygromycin B as selection agent to obtain cells stably expressing the c-Myc-D_2L_R. FlpIn CHO cells stably expressing the wild-type or mutant c-Myc-D_2L_R were grown in Dulbecco’s modified Eagle’s medium (DMEM) supplemented with 10% fetal bovine serum and 200 µg/mL of Hygromycin-B, and maintained at 37 °C in a humidified incubator containing 5% CO_2_. When cells were approximately 90% confluent, they were harvested and centrifuged (300 g, 3 min, 4 °C). The resulting pellet was resuspended in assay buffer either in the presence (20 mM HEPES, 100 mM NaCl, 6 mM MgCl_2_, 1 mM EGTA, and 1 mM EDTA, pH 7.4) or absence (20 mM HEPES, 100 mM NMDG, 6 mM MgCl_2_, 1 mM EGTA, and 1 mM EDTA, pH 7.4) of sodium ions. The centrifugation procedure was repeated. The intact cell pellet was then resuspended in assay buffer and homogenized using a Polytron homogenizer for three 10 s intervals on the maximum setting, with 30 s periods on ice between each burst. The homogenate volume was brought up to 30 mL, the sample was centrifuged (350 g, 5 min, 4 °C), the pellet was discarded, and the supernatant was centrifuged at 30 000 g for 1 hr at 4 °C. The resulting pellet was resuspended in 5 mL of assay buffer, and the protein content was determined using the bicinchoninic acid assay (BCA assay). The homogenate was then separated into 500 µl aliquots and stored at −80 °C until it was required for binding assays.

### [^3^H]spiperone binding assay

For saturating binding assays cell membranes (c-Myc-D_2L_-Flp-In CHO, 20 µg) were incubated with varying concentrations of [^3^H]spiperone and 10 µM haloperidol as a non-specific control, in binding buffer with (20 mM HEPES, 100 mM NaCl, 6 mM MgCl_2_, 1 mM EGTA, and 1 mM EDTA, pH 7.4) or without (20 mM HEPES, 100 mM NMDG, 6 mM MgCl_2_, 1 mM EGTA, and 1 mM EDTA, pH 7.4) sodium ions to a final volume of 1 mL and were incubated at 37 °C for 3 h. For competition and interaction binding assays cell membranes (c-Myc-D_2L_-Flp-In CHO, 20 µg) were incubated with varying concentrations of test compound in binding buffer with (20 mM HEPES, 100 mM NaCl, 6 mM MgCl_2_, 1 mM EGTA, and 1 mM EDTA, pH 7.4) or without (20 mM HEPES, 100 mM NMDG, 6 mM MgCl_2_, 1 mM EGTA, and 1 mM EDTA, pH 7.4) sodium ions, containing 0.15 nM of [^3^H]spiperone and 100 µM GppNHp to a final volume of 1 mL and were incubated at 37 °C for 3 h. Binding was terminated by fast-flow filtration over GF/B membranes using a Brandel harvester followed by three washes with ice-cold 0.9% NaCl. Bound radioactivity was measured in a Tri-Carb 2900TR liquid scintillation counter (PerkinElmer).

### Data analysis

Competition binding curves between [^3^H]spiperone and dopamine in the absence and presence of SB269652 were best fit to a one-site model^33^:

1 $$Y=\,\frac{{B}_{max}[A]}{[A]+(\frac{{K}_{A}{K}_{B}}{\alpha [B]+{K}_{B}})(1+\,\frac{[I]}{{K}_{I}}+\,\frac{[B]}{{K}_{B}}+\,\frac{{\alpha }^{\text{'}}[I][B]}{{K}_{I}{K}_{B}}\,)}$$

where K_I_ is the equilibrium dissociation constant of dopamine.

Competition binding curves between [^3^H]spiperone and SB269652, MIPS1726, MIPS1217 and MIPS1500 could be fit to the allosteric ternary complex model using the following equation^34^:

2 $$Y=\frac{\frac{[A]}{{K}_{A}}}{\frac{[A]}{{K}_{A}}+(\frac{1+\,\frac{[B]}{{K}_{B}}}{1+\alpha \frac{[B]}{{K}_{B}}})}$$

Where Y is percentage (vehicle control) binding; [A] and [B] are the concentrations of [^3^H]spiperone and ligand of interest, respectively; *K*_A_ and *K*_B_ are the equilibrium dissociation constants of [^3^H]spiperone and ligand of interest, respectively; and α is the cooperativity between the ligand of interest and [^3^H]spiperone. Values of α > 1 denote positive cooperativity, values < 1 (but > 0) denote negative cooperativity and values = 1 denote neutral cooperativity.

The concentration of ligand that inhibited half of the [^3^H]spiperone binding (IC_50_) was determined using the following equation:

3 $$Y=\,\frac{{\rm{Bottom}}+({\rm{Top}}-{\rm{Bottom}})}{1+{10}^{({\rm{X}}-{{\rm{logIC}}}_{50}){n}_{{\rm{H}}}}}\,$$

Where Y denotes the percentage specific binding, Top and Bottom denote the maximal and minimal asymptotes, respectively, IC_50_ denotes the X-value when the response is midway between Bottom and Top, and *n*_H_ denotes the Hill slope factor. IC_50_ values obtained from the inhibition curves were converted to K_i_ values using the Cheng and Prusoff equation^35^.

### Molecular modelling and simulations

The binding mode of SB269652 and its derivatives in the D_2_R were investigated as described in our previous study^2^. Briefly, to acquire a reference binding mode for the tetrahydroisoquinoline (THIQ) core of SB269652 in the high-resolution structure of the D_3_R (Protein Data Bank code 3PBL^36^, THIQ core in the protonated form was first docked into the D_3_R structure with the induced-fit docking (IFD) protocol^37^ implemented in Schrödinger software (release 2016–1, Schrödinger, LLC: New York, NY), and the lowest MM/GBSA energy pose from the largest pose cluster was selected as the reference pose. Assuming similar binding modes of the THIQ moiety in the near-identical OBS of the D_3_R and D_2_R, the pose from the IFD trial with our previous equilibrated D_2_R model^36,38^ that is closest to the reference pose in the D_3_R structure was selected. The full-length SB269652 was then docked into the D_2_R model by a core-constrained IFD protocol^38^ with restraints on the heavy atoms (heavy-atom RMSD deviation <2.0 Å) of the selected pose of the THIQ core. Representative poses for the SB269652 derivatives, were acquired similarly by using the core-restrain IFD protocol, where restraints were applied on the THIQ core of the full-length SB269652, assuming that the THIQ core adopts a similar pose in SB269652 derivatives as well.

MD simulations of the D_2_R–ligand complexes were performed in the explicit water and 1-palmitoyl-2-oleoylphosphatidylcholine lipid bilayer solvent environment using Desmond MD system (version 4.5; D. E. Shaw Research, New York, NY) with the CHARMM36 protein force field^39–41^, CHARMM36 lipid force field^42^, and TIP3P water model. The ligand parameters were obtained from the GAAMP server^43^, with the initial force field based on CGenFF assigned by ParamChem^44^. The system charges were neutralized, and a solvent concentration of 150 mM NaCl was added. The average system size is ∼111,300 atoms. Each system was first minimized and then equilibrated with restraints on the ligand heavy atoms and protein backbone atoms, followed by an isothermal–isobaric simulation at 310 K with all atoms unrestrained, as described previously^45–47^. For each complex, we ran multiple trajectories (Table 2).

MD simulation systems for Na^+^-unbound conditions were generated by removing Na^+^ ions from WT trajectories and then re-neutralizing the overall charge of the system by removing appropriate number of Cl^−^ ions in the water milieu.

### MM/GBSA Energy Calculations

MM/GBSA energy calculations on the D_2_R WT Na^+^-bound and -unbound conditions were performed using protocol implemented in CHARMM (version 40b2)^48^ using the GBSW implicit solvent model^49^. For each condition, we extracted frames at 1.2 ns interval from the last 300 ns of the MD trajectories.

### Data Availability

The datasets generated during the current study are available from the corresponding author on reasonable request.

## Electronic supplementary material

Supplementary Information
