# Supplementary material for: The action of a negative allosteric modulator at the dopamine D2 receptor is dependent upon sodium ions
Source: Sci Rep. 2018 Jan 19;8:1208. doi: 10.1038/s41598-018-19642-1 (PMC5775417; doi:10.1038/s41598-018-19642-1)
Supplement: Supplementary file 1 — Supplementary Information [file 41598_2018_19642_MOESM1_ESM.pdf]

Supplementary Information: The action of a negative allosteric modulator at the dopamine D_2 receptor is dependent upon sodium ions.

Christopher J. Draper-Joyce[1#], Ravi Kumar Verma[3#], Mayako Michino[3,6], Jeremy Shonberg[2], Anitha Kopinathan[2], Carmen Klein Herenbrink[1], Peter J. Scammells[2], Ben Capuano[2], Ara M. Abramyan[3], David M. Thal[1], Jonathan A. Javitch[4,5], Arthur Christopoulos[1], Lei Shi[3]*, J. Robert Lane[1]*

Drug Discovery Biology[1], Dept. of Pharmacology[1] and Medicinal Chemistry[2], Monash Institute of Pharmaceutical Sciences, Monash University, 399 Royal Parade, Parkville, VIC 3052, Australia

[3]Computational Chemistry and Molecular Biophysics Unit, National Institute on Drug Abuse Intramural Research Program, National Institutes of Health, 333 Cassell Drive, Baltimore, Maryland 21224, United States

Departments of Psychiatry and Pharmacology[4], College of Physicians and Surgeons, Columbia University, and Division of Molecular Therapeutics[5], New York State Psychiatric Institute, New York, New York 10032, United States

[6]Current address: Tri-Institutional Therapeutics Discovery Institute, 413 E 69th St, New York, NY 10021, United States

[#]both authors contributed equally to this work.

***To whom correspondence should be addressed:**

Dr. Lei Shi, Computational Chemistry and Molecular Biophysics Unit, National Institute on Drug Abuse Intramural Research Program, National Institutes of Health, 333 Cassell Drive, Baltimore, Maryland 21224, United States. Tel: (443)740-2774, Email: lei.shi2@nih.gov

Dr. J. Robert Lane, Drug Discovery Biology, Monash Institute of Pharmaceutical Sciences, 399 Royal Pde, Parkville, VIC 3052, Australia. Tel: +61399039095, Email: rob.lane@monash.edu

Supplementary Methods

Compound Synthesis

N-((*trans*)-4-(2-(7-Cyano-3,4-dihydroisoquinolin-2(1*H*)-yl)ethyl)cyclohexyl)-7-fluoro-1*H*-indole-2-carboxamide (**5**, MIPS1868).

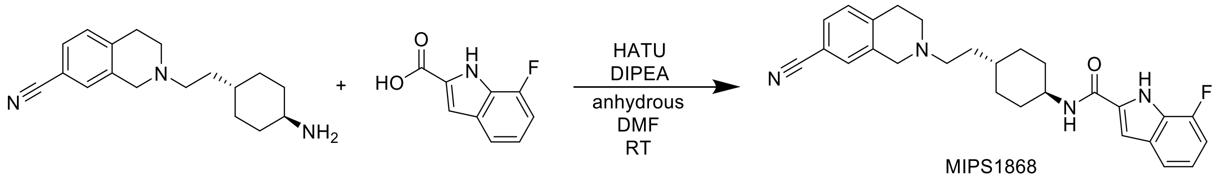

To a stirred solution of 2-(2-((*trans*)-4-aminocyclohexyl)ethyl)-1,2,3,4-tetrahydroisoquinoline-7-carbonitrile (126 mg, 444 μmol) and 7-fluoro-1*H*-indole-2-carboxylic acid (95.5 mg, 533 μmol) in a minimal volume of anhydrous DMF (3-4 mL) was added the coupling reagent, 1-[bis(dimethylamino)methylene]-1*H*-1,2,3-triazolo[4,5-*b*]pyridinium 3-oxid hexafluorophosphate (HATU, 253 mg, 666 μmol) and an excess of DIPEA. The reaction was stirred at room temperature overnight after which time, LCMS revealed complete consumption of starting material. The reaction mixture was then diluted with 30 mL of a 1:1 mixture of a saturated solution of $NaHCO_3$ and water and left to stir for a further 30 min. The resulting precipitate was filtered, washed with water, dried and then recrystallised via hot filtration in a mixture of MeOH and water to yield the title compound as a beige crystalline solid (112 mg, 57%). ^{1}H NMR (d_6-DMSO) δ 11.95 (s, 1H), 8.22 (d, J = 7.9 Hz, 1H), 7.59 – 7.54 (m, 2H), 7.45 – 7.40 (m, 1H), 7.31 (d, J = 8.4 Hz, 1H), 7.19 (d, J = 2.5 Hz, 1H), 7.04 – 6.96 (m, 2H), 3.75 (m, 1H), 3.57 (s, 2H), 2.88 (t, J = 5.6 Hz, 2H), 2.66 (t, J = 5.8 Hz, 2H), 2.55 – 2.44 (m, 2H), 1.86 (m, 4H), 1.45 (dd, J = 14.5, 6.8 Hz, 2H), 1.40 – 1.23 (m, 3H), 1.06 (m, 2H). ^{13}C NMR (d_6-DMSO) δ 160.0 (C), 149.7 (d, $^1J_{CF}$ = 245.1 Hz, C), 141.2 (C), 137.2 (C), 134.0 (C), 131.3 (d, $^4J_{CF}$ = 5.7 Hz, C), 130.8 (CH), 130.1 (CH), 129.9 (CH), 125.0 (d, $^3J_{CF}$ = 13.4 Hz, C), 120.4 (d, $^4J_{CF}$ = 6.0 Hz, CH), 119.6 (C), 118.0 (d, $^4J_{CF}$ = 3.4 Hz, CH), 108.6 (C), 108.3 (d, $^3J_{CF}$ = 16.2 Hz, CH), 104.6 (CH), 55.8 (CH_2), 55.3 (CH_2), 50.3 (CH_2), 48.8 (CH), 35.2 (CH), 34.0 (CH_2), 32.7 (CH_2), 32.1 (CH_2), 29.4 (CH_2).HPLC: t_R 6.36 min, >95% purity (λ = 214 & 254 nm). HRMS (*m/z*): $C_{27}H_{30}FN_4O$ requires $[M+H]^+$ 445.2404; found 445.2405.

N-((*trans*)-4-(2-(7-Cyano-3,4-dihydroisoquinolin-2(1*H*)-yl)ethyl)cyclohexyl)-1*H*-pyrrolo[2,3-*c*]pyridine-2-carboxamide (**4**, MIPS1726).

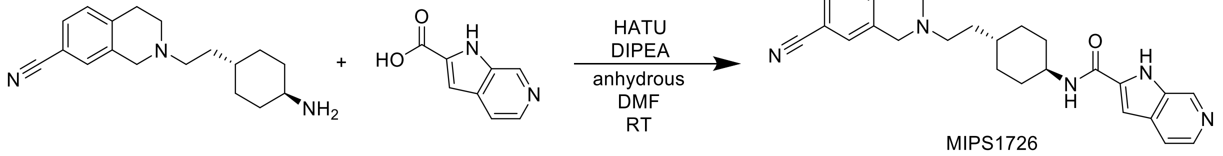

To a stirred solution of 2-(2-((*trans*)-4-aminocyclohexyl)ethyl)-1,2,3,4-tetrahydroisoquinoline-7-carbonitrile (29.2 mg, 103 µmol) and 1*H*-pyrrolo[2,3-*c*]pyridine- 2-carboxylic acid (33.4 mg, 206 µmol) in a minimal volume of anhydrous DMF (3-4 mL) was added the coupling reagent, 1-[bis(dimethylamino)methylene]-1*H*-1,2,3-triazolo[4,5-*b*]pyridinium 3-oxid hexafluorophosphate (HATU, 78.2 mg, 206 µmol) and an excess of DIPEA. The reaction was stirred at room temperature overnight after which time, LCMS revealed complete consumption of starting material. Prior to work up and purification, a small amount of precipitate was noted, which was filtered under vacuum and discarded. The filtrate was diluted with 30 mL of a 1:1 mixture of a saturated solution of $NaHCO_3$ and water and then extracted using EtOAc (3 × 20 mL). The organic layers were pooled, washed with water (2 × 20 mL) and brine (20 mL) and dried over anhydrous Na_2SO_4. The mixture was then evaporated to dryness and purified by flash chromatography (10% MeOH/ DCM) to produce the title compound as an off-white, waxy residue (18 mg, 41%). ^{1}H NMR (MeOD) δ 8.75 (br s, 1H,), 8.08 (br s, 1H), 7.63 (m, 1H), 7.46 (m, 2H), 7.28 (d, *J* = 8.5 Hz, 1H), 7.12 (s, 1H), 3.85 (m, 1H), 3.73 (s, 2H), 2.98 (m, 2H), 2.90 – 2.80 (m, 2H), 2.64 (m, 2H), 2.02-1.81 (m, 4H), 1.63 – 1.51 (m, 2H), 1.51 – 1.28 (m, 3H), 1.28 – 1.05 (m, 2H). ^{13}C NMR (MeOD) δ 210.5 (C), 162.2 (C), 141.3 (C), 138.2 (CH), 137.4 (C), 136.7 (C), 136.0 (CH), 134.2 (C), 131.7 (CH), 131.1 (CH), 130.9 (CH), 119.9 (C), 110.7 (C), 102.9 (CH), 57.0 (CH_2), 56.0 (CH_2), 51.3 (CH_2), 50.6 (CH), 36.6 (CH), 34.4 (CH_2), 33.4 (CH_2), 33.1 (CH_2), 30.7 (CH), 29.5 (CH_2). HPLC: t_R 4.45 min, >95% purity (λ = 214 & 254 nm). HRMS (*m/z*): $C_{26}H_{30}N_5O$ requires $[M+H]^+$ 428.2450; found 428.2457.

Supplementary Results

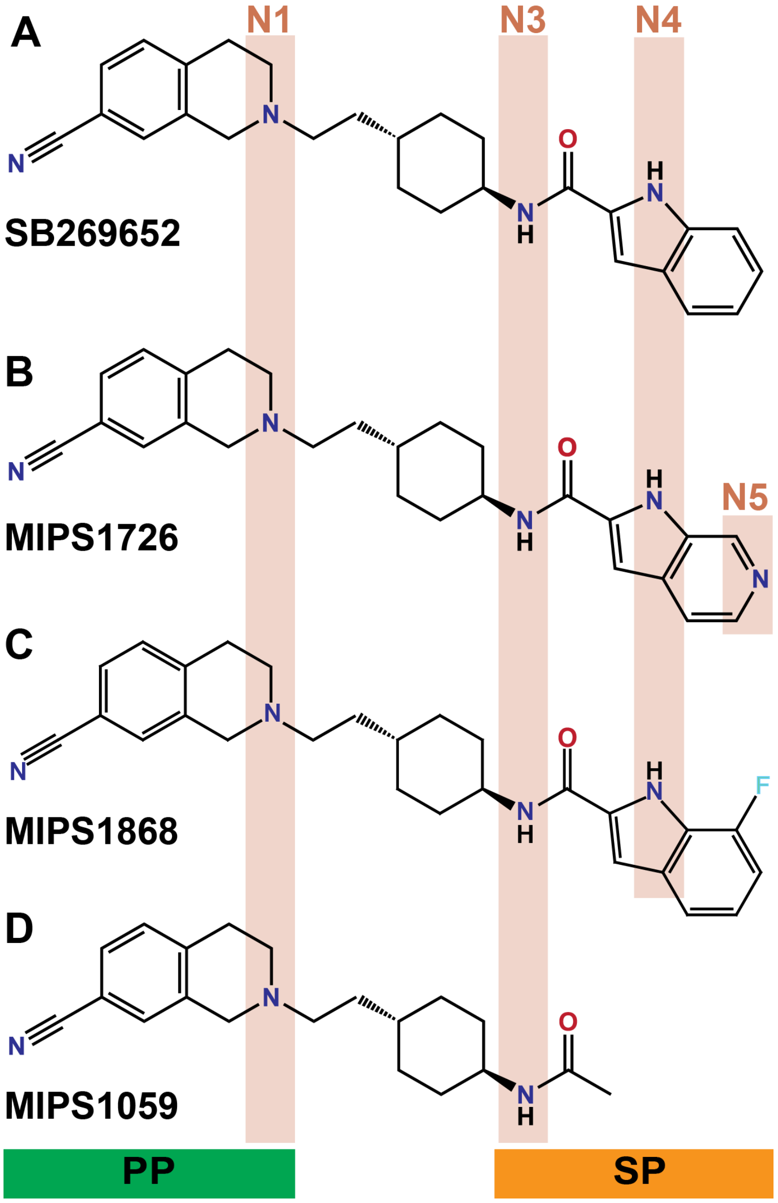

Supplementary Figure 1. The structure of SB269652 and its derivatives in which the 7-cyano-tetrahydroisoquinoline moiety is defined as the primary pharmacophore (PP) and the aryl amide moiety is defined as the secondary pharmacophore (SP). The nomenclature and positions of ligand nitrogen atoms (N1, N3, N4, and N5) used in our modelling analysis are indicated.

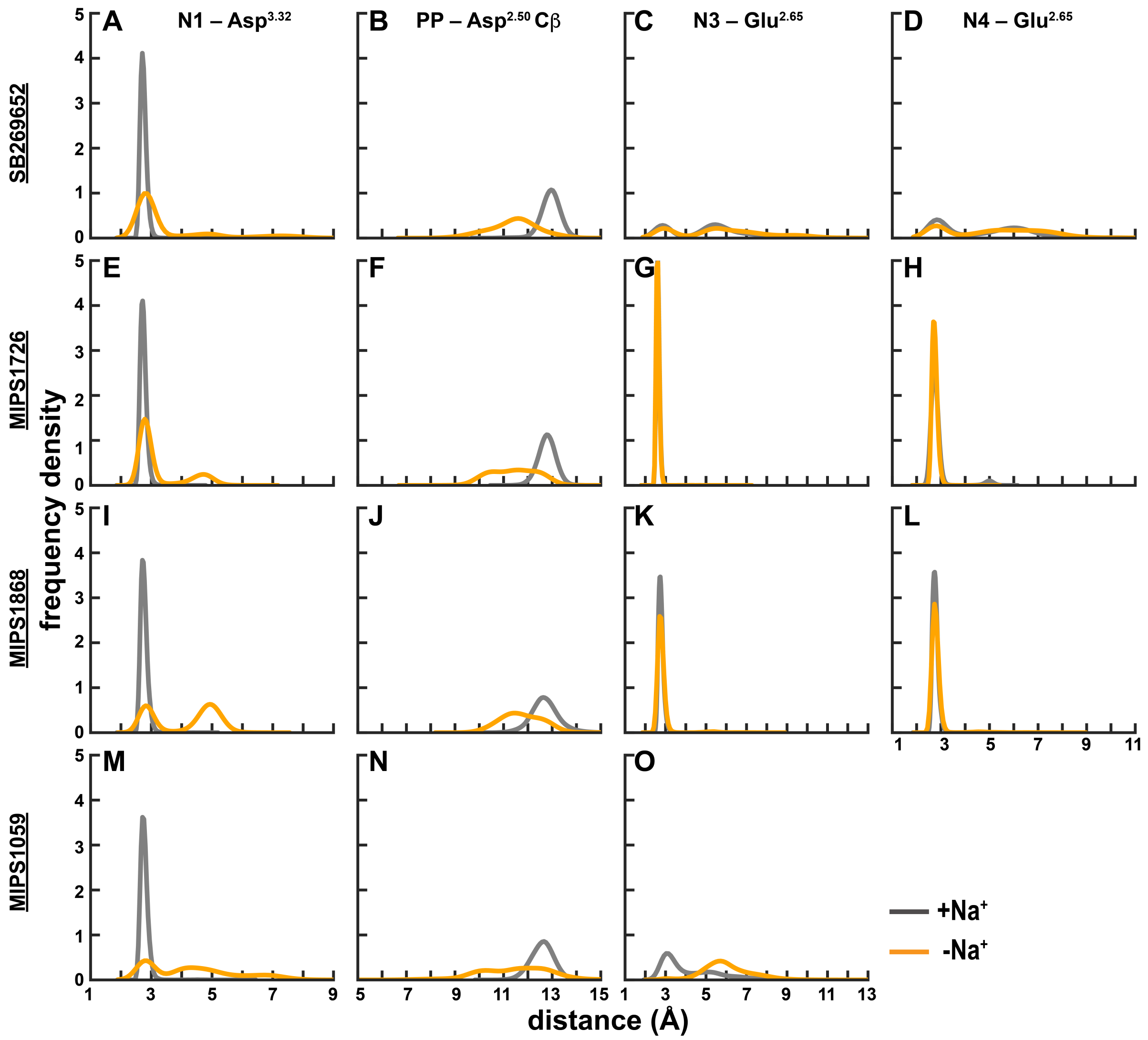

Supplementary Figure 2. Key distances between the ligands and the receptor. The Na^+-bound (+Na^+) and -unbound (-Na^+) conditions for each ligand are shown in grey and orange, respectively. Panels **A**, **E**, **I**, and **M** show distribution of the minimum distance between the protonated N1 atom of the ligands (see Supplementary Figure 1) and the carboxyl oxygen atoms of Asp114$^{3.32}$ in each condition. Note in -Na^+ condition this interaction significantly weakens for all these ligands. In panels **B**, **F**, **J**, and **N**, smaller minimum distances between the ligand PP and the Cβ atom of Asp80$^{2.50}$ in the -Na^+ condition indicate downward movements of the PP. Panels **C**, **D**, **G**, **H**, **K**, **L** and **O** show that MIPS1726 and MIPS1868 have significantly stronger interactions between ligand N3 or N4 atoms and the carboxyl oxygen atoms of Glu95$^{2.65}$.

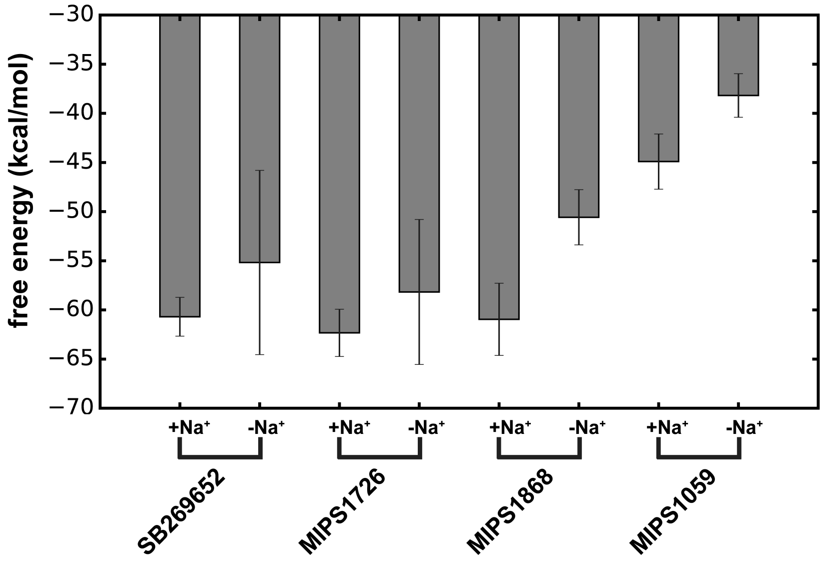

Supplementary figure 3. The binding free energies estimated by MM/GBSA calculations indicate consistent favored binding poses in the presence of Na^+.

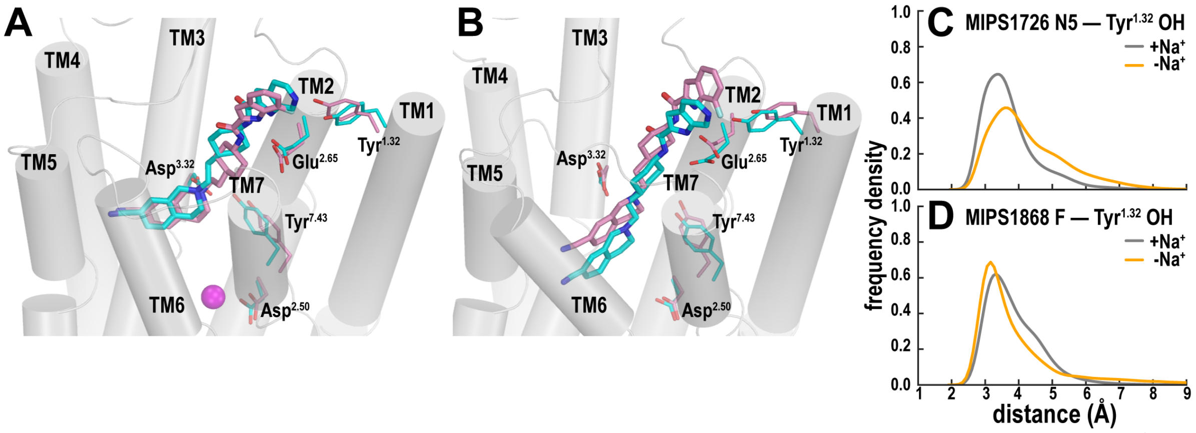

Supplementary Figure 4. Comparisons of MIPS1868 and MIPS1726 poses in the Na^+ bound (+Na^+) and -unbound (-Na^+) conditions. (A) Similar binding poses of MIPS1868 (pink) and MIPS1726 (cyan) in the +Na^+ condition. (**B**) The aryl moiety of MIPS1868 (pink) is positioned more extracellularly than that of MIPS1726 (cyan) in the -Na^+ condition. (**C**) MIPS1726 has lower interaction frequencies with $Tyr34^{1.32}$ sidechain hydroxyl group in the -Na^+ condition than in the +Na^+ condition, whereas (**D**) MIPS1868 has similar interaction strengths in both conditions.

Supplementary Table 1: Comparison of the difference in affinity of various SB269652 derivatives and fragments at the WT and $E95^{2.65}$A $D_{2L}R$ in the presence and absence of Na^+. Values are the difference between the affinity (pK_B) of the compound at the WT and $E95^{2.65}$A $D_{2L}R$ in the presence and absence of Na^+. Values represent the mean ± S.E.M. of three experiments performed in duplicate, values in brackets represent fold difference in pK_B between WT and $E95^{2.65}$A. *Statistically different from corresponding value in the presence of Na^+ ($p < 0.05$, Students unpaired two-tailed t-test).

	**ΔpK_B (WT-$E95^{2.65}$A)**	
**Compound**	**+Na^+**	**-Na^+**
**MIPS1059**	0.71 ± 0.09 (5-fold)	0.44 ± 0.12* (3-fold)
**MIPS1726**	0.61 ± 0.07 (4-fold)	0.31 ± 0.10* (2-fold)
**MIPS1868**	0.33 ± 0.09 (2-fold)	0.00 ± 0.11* (no difference)
